# The effects of suicidal ideation and constructs of theory of planned behavior on suicidal intention in women: a structural equation modeling approach

**DOI:** 10.1186/s12888-020-02625-w

**Published:** 2020-05-11

**Authors:** Forouzan Rezapur-Shahkolai, Mehdi Khezeli, Seyyed-Mohammad-Mahdi Hazavehei, Saeed Ariapooran, Ali Reza Soltanian, Alireza Ahmadi

**Affiliations:** 1 Department of Public Health, School of Public Health, Hamadan University of Medical Sciences, Hamadan, Iran; 2 Social Determinants of Health Research Center, Hamadan University of Medical Sciences, Hamadan, Iran; 3 Social Development and Health Promotion Research Center, Health Institute, Kermanshah University of Medical Sciences, Kermanshah, Iran; 4 Research Center for Health Sciences, Hamadan University of Medical Sciences, Hamadan, Iran; 5 Department of Psychology, Malayer University, Malayer, Iran; 6 Department of Biostatistics, School of Public Health, Hamadan University of Medical Sciences, Hamadan, Iran; 7 Modeling of Non-communicable Diseases Research Center, Hamadan University of Medical Sciences, Hamadan, Iran; 8 Department of Anesthesiology, Imam Reza Hospital Center, Kermanshah University of Medical Sciences, Kermanshah, Iran

**Keywords:** Mental health, Suicidal thought, Theory of planned behavior, Women

## Abstract

**Background:**

The Theory of Planned Behavior (TPB) is proposed to predict behavioral intention. We conducted this study aimed to investigate the effects of Suicidal Ideation (SI) and constructs of TPB on suicidal intention.

**Methods:**

This cross-sectional study was conducted with 923 married women selected by multi-stage sampling method from Gilan-e Gharb County, the west part of Iran, in 2018. Data gathering tools were a questionnaire on demographic characteristics, the Beck Scale for Suicidal Ideation, and a four-part questionnaire based on constructs of TPB, including Attitude, Subjective Norms, Perceived Behavioral Control (PBC), and Intention. Data were analyzed by SPSS 19 and AMOS using Structural Equation Modeling (SEM).

**Results:**

Out of 923 participants, 345 women (37.4%) had some degree of suicidal ideation. The mean score of suicidal ideation in all of participants was 5.98 ± 7.79, while in the 345 individuals with suicidal ideation was 15.53 ± 3.65. Suicidal ideation had the strongest positive total effect on suicide intent, while PBC and attitude had the negative effect on suicide intent.

**Conclusions:**

Since suicidal ideation had the strongest direct effect on suicidal intent, it is suggested that this variable be used for risk assessment in all suicide prevention programs and counseling measures be implemented to reduce suicidal thoughts. Also, undesirable attitudes toward suicide and perceived behavioral control on suicide intention can be considered and emphasized in planning future interventions.

## Background

Worldwide, suicide accounts for nearly 800,000 deaths every year, with a global age-standardized rate of 10.5 per 100,000 population [1]. Suicide is known to be influenced by a variety of psychological, social, economic, political and religious factors in different populations with different geographical and cultural contexts [2]. The rate of suicide is two to five times higher in the western provinces of Iran than the average rate of the country [3]. Kermanshah province in the west of Iran had a high rate of mortality caused by suicide. Also, Gilan-e-Gharb County located in the west of Kermanshah province has a high rate of attempted suicide among women in province [4]. The findings of a study showed that 48% of bereaved women of Gilan- gharb had some degrees of suicidal ideation [5]. Suicidal ideation is the thoughts and fantasies about killing themselves, which can included a range of quick thoughts about the death to complete preoccupation with self-destruction [6]. Suicidal intent is one of the other important factors contributing to suicide and defined as the desire for death and suicide attempt. The degree of suicide intention can predict the method used and the lethality of suicide attempts [7]. Results of a qualitative study in women of Gilan-e gharb who experienced a suicide attempt revealed that both individual and social factors are responsible for attempting suicide, and suicidal ideation and intention have played a decisive role on suicidal behavior [8]. It seems that people who choose lethal methods to attempt suicide have a real intention to end their lives [9]. The Theory of Planned Behavior (TPB), posits that proximal predictor of any behavior is ones behavioral intention that means ones motivation to engage in the behavior [10]. Suggested by Ajzen and Fishbein [11], intention is determined by Attitudes (overall positive and negative evaluations of behavior), Subjective Norms (perceived social pressure from others), and Perceived Behavior Control related to one’s control over performing the behavior. A previous study has suggested that the level of suicidal intent is a powerful predictor of death from attempted suicide [12]. Current intent for suicide is a key component of suicide risk assessment protocols and is conceptualized as a necessary component of serious and imminent risk for suicide [13]. A relevant number of studies has investigated the relationship between suicidal ideation and suicide attempt, while the relationship between suicidal ideation and suicide intent less studied [14]. Few previous studies have shown that TPB is a useful framework for assessing suicide behavior, especially suicide intent [14–16].

In the present study, we aimed to investigate the effects of suicidal ideation and TPB constructs on suicidal intention. Main research hypothesis was that suicidal ideation has the significant effect on suicide intent and can play a mediation role between constructs of TPB - attitude, subjective norms, and perceived behavioral control – and suicide intention. Another hypothesis was that constructs of TPB have significant effects on suicide intention.

## Methods

### Sample and procedure

The sample conceived for this study was calculated as 1037 people, on the basis of the appropriate sample size formula and considering an effect size of 1.2. As a matter of fact, in total, 923 women completed the data gathering tools, demonstrating a response rate of 89%. This cross-sectional study was conducted in 2018 on married women aged 18 to 59 years from Gilan-e Gharb County, located in the west part of Iran. We selected women as the sample of the study because suicide attempt in women is significantly more than men in Kermanshah province and especially in Gilan Gharb city [4, 17]. Married women were also selected because all of them had a health dossier in healthy centers and access to them was easier for health experts and researchers. It should be noted that this article was a part of a larger project aimed at reducing suicide intentions in women of Gilan-e gharb, therefore access to the subjects was an important for the research team. Inclusion criteria were residence in Gilan-e-Gharb County, not having severe mental and physical illness, being married, and being between the ages of 18 to 59. An exclusion criterion was also incomplete questionnaire. Participants were selected using multi-stage sampling method, according to which, a list of women who had a health dossier in all of six healthy centers of Gilan-e Gharb was prepared. Then, according to the population of each center, the number of women according to the calculated quota was randomly selected from the list. After explaining the purpose of research to the participants, informed consent form was obtained and participants completed the questionnaire, which lasted about 45 min. Data were collected from June to August 2018.

### Measures

In this study, data were collected using the demographic information form, Beck Scale for Suicidal Ideation (BSSI), and TPB Questionnaire.

#### Beck scale for suicidal ideation (BSSI)

This scale measures the severity of suicidal ideation using 19 items; each rated from 0 to 2. The total scores on the BSSI can thus range from 0 to 38 points, in which higher scores indicate the more intense suicidality. Since there is no specific cut-off score in BSSI [18], we used the Beck and Steer’s method of screening for suicide ideators on the BSSI [18, 19], according to which fourth or fifth questions must receive a rating greater than zero. Accordingly, the first five questions were used to screen for attitudes toward living and dying, and only patients who reported a desire to make an active or passive suicide attempt (questions four and five), were allowed to answer the other questions of BSSI. These items have already been introduced as screening items in the questionnaire [18, 20]. Anisi et al. performed semantic, technical, and criterion equivalence by translating and back translating the instrument into Persian language. The concurrent validity of the scale with the General Health Questionnaire has been reported as 76% and internal consistency using Cronbach’s alpha was 0.95 [21]. In the present study, Cronbach’s alpha coefficient for internal consistency of suicidal ideation questionnaire was 0.967.

#### Theory of planned behavior questionnaire

This questionnaire included 26 questions in four sections based on the constructs of TPB including Attitude, Subjective Norms, Perceived Behavioral Control, and Intention. Attitude was measured by nine questions, for example: “Suicide attempt is a definite way to escape from the unsolved problems of life”. Subjective Norms subscale had four questions, i.e., “My husband’s opinion about suicide is important to me”. Perceived Behavioral Control was measured by nine questions, for example: “Even if I have severe conflict with my husband, I can control the inclination for attempting suicide”. Finally, intention was assessed using four questions, i.e., “I intend to die by suicide in the future and I’ve also planned it”. All questions were scored on a 5-point Likert scale ranged from 1 (‘strongly disagree’) to 5 (‘strongly agree’). The content validity of questionnaire was measured using content validity ratio (CVR), and content validity index (CVI) via an experts’ panel including eight health education and promotion specialists, two psychologists and one epidemiologist. Intra-class correlation coefficients of Attitude, Subjective Norms, Perceived Behavioral Control, and Intention subscales were 0.76, 0.84, 0.78, and 0.88, respectively. In addition, using Cronbach’s alpha, internal consistency were 0.87, 0.82, 0.92, and 0.83 respectively.

### Ethical aspect of the study

Confidentiality of information was guaranteed to all participants, and written informed consent form was obtained from all of them. This study received ethics approval from the Research Ethics Committee of Hamadan University of Medical Sciences (No.IR.UMSHA.REC.1395.45) and Kermanshah University of Medical Sciences (No.IR.KUMS.REC.1395.506).

### Analysis

Analyses were done by transferring the data to SPSS21, and AMOS software. Descriptive statistics are presented as Mean ± SD, or rate (%), for the evaluation of socio-demographic data and baseline variables of the participants. Structural Equation Modeling (SEM) was conducted in AMOS software. Using SEM, goodness of fit and also the significance of variables effects were investigated. For this purpose we used indices such as Chi-square Mean/Degree of Freedom (CMIN/DF), Root Mean Square Error of Approximation (RMSEA), Comparative Fit Index (CFI), Goodness of Fit Index (GFI) and Adjusted Goodness of Fit Index (AGFI). Also, using bootstrap estimates, the direct, indirect, and total effects of variables and their significance were assessed. In this study, the dependent variable was suicide intention, and the independent variables were suicidal ideation, attitudes, subjective norms, and perceived behavioral control.

## Results

In the present study, 1037 women were eligible to participate in the study that ultimately 923 completed the questionnaire and participated in the study with mean age of 37.28 ± 9.58 years. Table 1 showed the demographic characteristics of the participants. According to this table, the majority of subjects were housewives (84.7%). In terms of formal education more than half of the people did not attend high school (57.6%). At the time of the study, 5.4% of women were pregnant. In this study, out of 923 participants, 345 women (37.4%) had some degree of suicidal ideation. Friends/relatives history of attempted suicide, family history of attempted suicide, and history of attempted suicide in participants were 34.9, 16.1, and 11.9% respectively. More details about demographic characteristics of the subjects are presented in Table 1.

**Table 1:** Frequency distribution of the demographic characteristics of participants (*n* = 923)

Variables	Category	N (%)
Educations	Illiterate	125 (13.5)
	Preliminary	135 (14.6)
	intermediate	272 (29.5)
	High school diploma	191 (20.7)
	Academic education	200 (21.7)
Employment	employed	113 (12.2)
	worker	8 (.9)
	free job	18 (2.2)
	housewife	782 (84.7)
Friends/Relatives History of attempted suicide	Yes	322 (34.9)
	No	601 (65.1)
Family History of attempted suicide	Yes	149 (16.1)
	No	774 (83.9)
History of attempted suicide	Yes	110 (11.9)
	No	813 (88.1)
Having suicidal ideation	Yes	345 (37.4)
	No	578 (62.6)

As shown in Table 2, the mean score of suicidal ideation in all of participants was 5.98 ± 7.79. The mean score of TPB constructs were also as follow: Attitude = 30.19 ± 4.32, Subjective norms = 12.89 ± 2.53, Perceived Behavioral Control = 28.04 ± 4.01, and Intention = 7.27 ± 2.75. Table 2 also showed the correlations of research variables in which suicidal ideation had the highest correlation with suicide intention (*r* = 0.671, *p* < 0.001).

**Table 2:** Correlation between the construct of TPB and suicidal ideation

Variables	Mean ± SD, (Median)	correlations				
		1	2	3	4	5
1. Intention	7.27 ± 2.75 (7)	1				
2. Suicidal ideation	5.98 ± 7.79 (0)	0.671^a^	1			
3. Attitude	30.19 ± 4.32 (30)	−0.469^a^	− 0.609^a^	1		
4. Subjective norms	12.89 ± 2.53 (13)	−.0398^a^	−0.561^a^	0.397^a^	1	
5. Perceived behavioral control	28.04 ± 4.01 (28)	−0.436^a^	−0.471^a^	0.437^a^	0.368^a^	1

^a^Significant at 0.05 level

It should be noted that before running the model, the probability of collinearity between the suicidal intention and dependent variables was investigated and the results of tolerance factor and the variance inflation factor (VIF) showed that there is no Multicollinearity between the variables. Also, because the variables did not follow the normal distribution, the bootstrap test was used to examine the significance of the effect of variables.

According to the Model Fit Summary presented in Table 3, the Absolute Fit Indices of research model included the Goodness of Fit Index (GFI) = 0.885, and Adjusted Goodness-Fit Index (AGFI) = 0.874; these appear at acceptable level. The Parsimonious Fit Indices included the Chi-square Statistics (CMIN/DF) ratio = 3.61, and the Root Mean Square Error of Approximation (RMSEA) = 0.072, which are also acceptable. This holds true also for the Comparative Fit Index (CFI) = 0.885.

**Table 3:** Model fit summary of Theory of Planned Behavior for suicidal intention

Index name	acceptable fit ranges	good fit ranges	Observed index	Result
(CMIN/DF) Value	–	–	2765.32 / (765)	Acceptable fit
*P*-value	–	–	< 0.001	
(CMIN/DF) ratio	< 5	< 3	3.61	
RMSEA	< 0.08	< 0.05	0.072	Acceptable fit
CFI	> 0.90	> 0.95	0.908	Acceptable fit
GFI	> 0.85	> 0.90	0.885	Acceptable fit
AGFI	> 0.85	> 0.90	0.874	Acceptable fit

*CMIN/DF* Chi-Square Mean/Degree of Freedom, *RMSEA* Root Mean Square Error of Approximation, *CFI* Comparative Fit Index, *GFI* Goodness of Fit Index, *AGFI* Adjusted Goodness of Fit Index

**Fig. 1:**
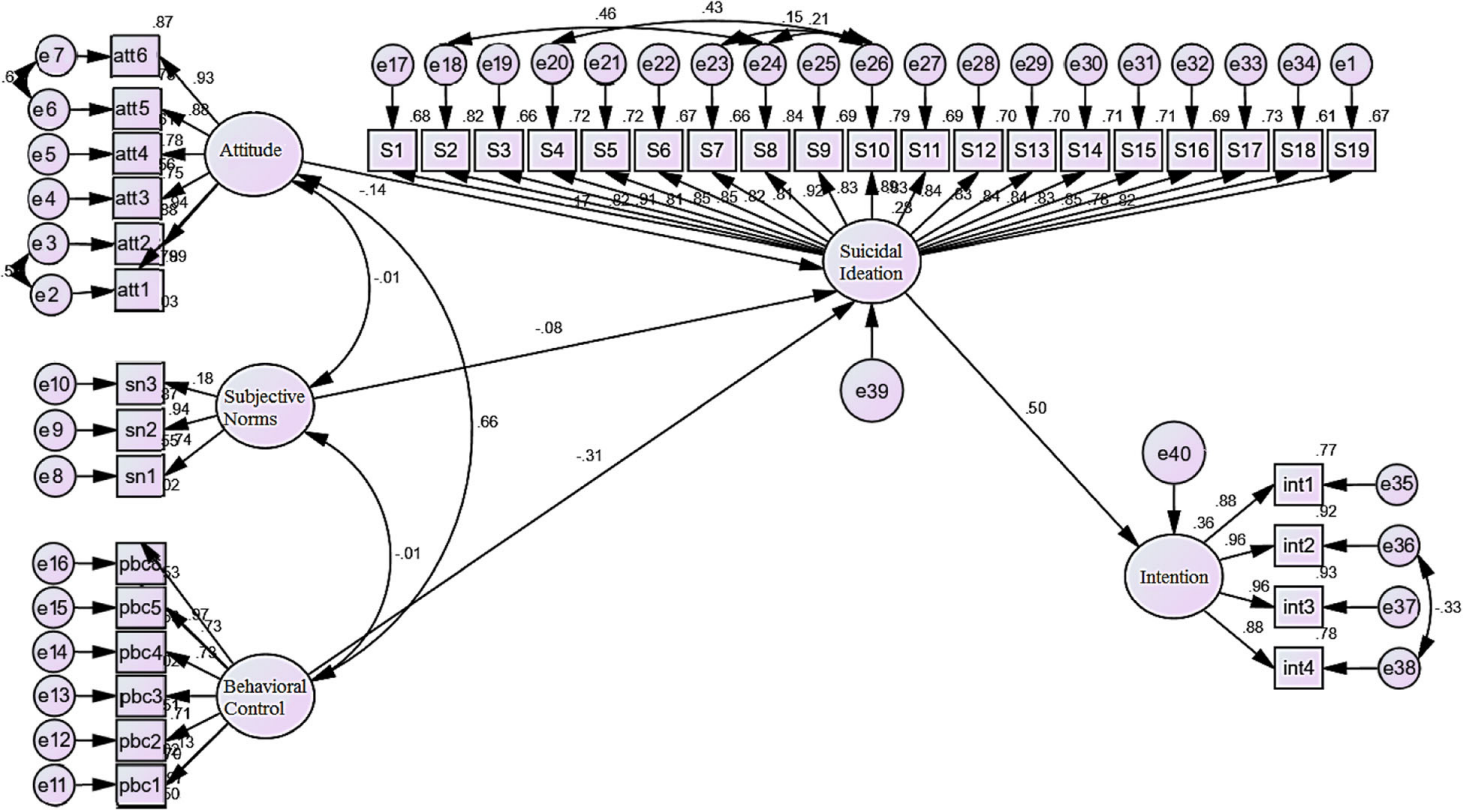
Standard factor loading of research model (Theory of Planned Behavior); significant direct effect of attitude = − 0.14, and perceived behavioral control = − 0.31 on suicidal ideation, and direct effect of suicidal ideation = 0.50 on suicidal intention

Table 4 and Fig. 1 shows the factor load and significance of the test. According to the results of SEM, the effect of PBC (*p* < 0.001) and attitude (*p* = 0.043) on suicidal ideation were significant (negative and inverse), but the effect of subjective norms was not significant (*p* > .05). Also the effect of suicidal ideation on intention was significant (*p* < 0.001), positive and direct.

**Table 4:** The result of running the TPB for suicidal intention using the structural equation model

Paths			unstandardized factor loads	standardized factor loads	Stimation error	t-value	*p*-value
**ATT**	➔	**INT**	−0.130	−0.14	0.023	−4.249	=.043
**SN**	➔		−0.081	−0.08	0.024	−1.270	=.18
**PBC**	➔		−0.272	−0.31	.022	−11.400	<.001
**SI**	➔		0.887	0.50	0.047	18.728	<.001

*ATT* Attitude, *SN* Subjective Norms, *PBC* Perceived Behavioral Control, *SI* Suicidal Ideation, *INT* Intention

Table 5 shows the direct, indirect and total standard effects of independent variables on the suicidal intention using the repetition in Bootstrap method assuming 5000 samples. According to the results, the direct effects of perceived behavioral control (PBC), attitude (ATT) and suicidal ideation (SI) on the suicidal intention (INT) were significant and inverse, inverse, and direct, respectively. The strongest total effect belonged to the suicidal ideation (SI); also PBC and attitude had the highest effects in negative direction. The effect of subjective norms on suicidal intention was not significant.

**Table 5:** Direct, indirect and total standardized effects of independent variables on suicidal intention using Bootstrap method: application of TPB

Paths			direct effect (bootstrap estimate error)	p	Indirect effect (bootstrap estimate error)	p	total effect (bootstrap estimate error)	P
**ATT**	➔	**INT**	–	–	−0.014 (0.008)	=0.038	−0.014 (0.008)	=0.038
**SN**	➔		–	–	−0.007 (0.006)	=.222	−0.007 (0.006)	=.222
**PBC**	➔		–	–	−0.113 (0.008)	<.001	−0.113 (0.008)	<.001
**SI**	➔		0.372 (0.011)	<.001	–	–	0.272 (0.011)	<.001

*ATT* Attitude, *SN* Subjective Norms, *PBC* Perceived Behavioral Control, *SI* Suicidal Ideation, *INT* Intention

## Discussion

In the present study, more than one-third of the women had some degree of suicidal ideation. The results of SEM showed that suicidal ideation, PBC, and attitude toward suicide had significant effect on suicidal intention, while subjective norms had not a significant role.

As earlier mentioned, in this study, 37.4% of women reported some degree of suicidal ideation. The results of a cross-national study conducted in 17 countries from different parts of the world showed that lifetime prevalence of suicidal ideation was 9.2% [22]. Two studies conducted in central and south parts of Iran reported suicidal ideation of 12.8 and 10% in women [23, 24]. The result of present study compared to other studies conducted in Iran and other parts of the world indicates that the prevalence of suicidal ideation in our sample is considerable [3, 23, 25]. Generally, many studies have shown that suicidal ideation in women is higher than in men. For example a community surveys in 21 countries estimated that the 12-month prevalence of suicidal ideation was greater in females than men [26].

In the present study, we used the TPB as a platform and conceptual framework of the study in which the suicidal ideation variable was added. The Theory of Planned Behavior is proposed to predict behavioral intent [27]. In fact, this theory cannot directly predict how much a person is likely to commit suicide. TPB suggests that attitudes, subjective norms and perceived behavioral control can predict behavioral intent [15]. In addition to these variables, we also assessed the role of suicidal ideation on suicidal intent, according to a study by George [14]. Ajzen proposed that the TPB can be utilized to understanding the individuals’ levels of suicidal ideation and intent [27]. Several studies have suggested that we need to move beyond clinical and demographic factors to further understanding of suicide [28]. As suggested by George, researchers must merge approaches to assessing suicidal intent, such as integrating identified variables in past researches with social and cognitive influences [14]. Utilizing the TPB can help promote understanding of suicidal ideation and behavior in regard to attitude and outcome beliefs associated with engaging in suicidal behavior, pressure or influence of important others, and perceived control in ability to overcoming suicide intent. In this study, using the TPB integrated with suicidal ideation we attempted to evaluate the effects of psychological and social factors limited to the TPB framework on suicide intention.

The results showed that suicidal ideation had a significant powerful effect on suicidal intention. Finding of a study showed that current suicidal ideation accounted for 56% of the variance in current suicidal intent [20]. The continuum of suicidal behavior includes death wishes, suicidal ideation, suicidal attempt, and suicide [29]. It is also known that suicidal ideation is the best predictor of an attempt and subsequently attempt is a predictor of suicide [30].. Beck and Weishaar identified suicidal ideation and intent as significant predictors of suicide [31]. Attempters who show persistent suicidal ideation with high intent to die are at high risk of re-attempting suicide [32].

The results showed that PBC and attitude after suicidal ideation had significant effect on suicidal intention, but subjective norms did not play any significant effect. The results of a study showed that attitudes towards suicide, subjective norms, and perceived behavioral control accounted for 49% of the variance in current suicidal ideation, where PBC accounted for 43% of the variance, attitude explained an additional 6% of the variance, and subjective norms regarding suicide accounted for less than 1% of the variance [14]. In one study, extended TPB variables explained almost 50% of the variance associated with intention to deliberately self-harm [16]. In the mentioned study, self-efficacy, attitude, and moral norms were significant predictors of suicidal intention while subjective norms and anticipated affect did not play a role in predicting suicidal intention [16]. Matheson found that PBC, attitudes toward suicide, and subjective norms, accounted for 72% of the variance related to suicidal intent. Although all three constructs of the TPB were statistically significant, the greatest proportion was for PBC, while subjective norms played a very small role in predicting the suicide intent [15]. PBC in this study consistent with other studies had a significant effect on suicidal intent [14–16], however adding suicidal ideation as strongest predictor of suicidal intent to TPB the role of PBC in predicting suicide intent was reduced. A study showed that attitude to permissiveness of suicide attempts were significantly higher in women than men, according to which females were about three times as likely as males to report permissive attitudes [33]. Literature refers to a phenomenon called gender paradox in suicide according to which men have a lower rates of suicidal ideation, but use more lethal suicide methods and kill themselves three to four times more than women whereas women reveal greater levels of suicidal ideation and of suicide attempts [34]. Evidence shows that women who attempt suicide are less likely to die, so they usually choose methods with less lethality. It depends on people’s attitude toward suicide [35–37]. According to a qualitative study women attempt suicide for reasons other than death, and from their point of view, suicide is a means for escaping from problems or meeting hidden desires [8]. There is also evidence that people who is more accepting suicide, exhibit also higher levels of suicidal ideation [38]. Despite this, remarkably few studies have focused on suicidal behavior in women or tried to explore the complex relationships between gender and suicidal behavior [39]. One reason for lowering suicide investments in women is probably that official statistics in most countries have focused on suicide deaths and ignored suicidal thoughts and intentions that are more prevalent in women [40]. Therefore, studies such as the present study that investigate the suicide intention and related factors such as attitude toward suicide in women can provide appropriate knowledge about the status of suicide intention in women.

In the present study, subjective norms had no significant role in predicting suicide intent. To examine the subjective norm, participants were asked to rate the impact of the views of the four groups - friends, spouse, family members, and religious leaders - on their suicidal ideation. In case of suicide, subjective norm based on few studies conducted using the TPB played a small role in predicting suicide intent [14–16].

This study had some limitations that need to be considered when interpreting the findings of the study. This study was conducted only on women, which makes it impossible to generalize the results to the men. Another limitation of the study was that the people who likely had the intention and decision to die but underestimated their intention to report in the present study. Also, in the present study we assessed only the impact of suicidal thoughts and constructs of theory of planned behavior including attitude, subjective norms and perceived behavioral control on suicide intention and the role of other contextual, individual and social factors were not examined. However, this study yielded significant results regarding the direct role of suicidal ideation on suicide intention. Also, the application of suicidal ideation in the TPB conceptual framework showed that by incorporating this variable into the theory, the prediction power of suicide intention more increased. However there are others strengths in the present study. First, we used a large sample size that can give more accurate estimates. Second, a very high response rate (89%) along with a randomly selected sample decreased the possibility of selection bias.

## Conclusion

This study concluded that suicidal ideation, PBC, and attitude toward suicide had significant effect on suicidal intention, while subjective norms did not play a significant role. According to the results of study, we suggest in addition to the existing follow-up and treatment protocols based on Mental Health Program Integrated into the Primary Health Care System of Iran, interventions could be developed to modify the suicidal ideation and intention of women toward suicide using Theory of Planned Behavior. Since, suicidal ideation had the strongest direct effect on suicidal intent, it is suggested that this variable be used for risk assessment in suicide prevention programs for women and counseling programs to reduce suicidal ideation be implemented. Given the effect of attitude on suicide intention in women according to the present study, it is suggested that studies on suicidal behaviors carefully evaluate this component, and intervention programs target strategies to reduce both individual and social attitudes toward suicide. For this purpose, consistent with other studies [41, 42], we suggest strategies that increase perceptions about disadvantages of suicide for the individual and the family, enhance the attitude toward help-seeking, treat depression and hopelessness, and reduce the social desirability of suicide among women. Also considering the role of perceived behavioral control, it is suggested that interventions that enhance life skills such as resilience and anger management be implemented for women.

## Data Availability

The data sets used and analyzed in this study are available from the corresponding author on reasonable request.

## Abbreviations

AGFI: Adjusted Goodness of Fit Index
ATT: Attitude
CFI: Comparative Fit Index
CMIN/DF: Chi-square Mean/Degree of Freedom
GFI: Goodness of Fit Index
INT: Suicidal Intention
PBC: Perceived Behavioral Control
RMSEA: Root Mean Square Error of Approximation
SEM: Structural Equation Modeling
SI: Suicidal Ideation
SN: Subjective Norms
TPB: Theory of Planned Behavior
